# On the SARS-CoV-2 “Variolation Hypothesis”: No Association Between Viral Load of Index Cases and COVID-19 Severity of Secondary Cases

**DOI:** 10.3389/fmicb.2021.646679

**Published:** 2021-03-16

**Authors:** Mattia Trunfio, Bianca Maria Longo, Francesca Alladio, Francesco Venuti, Francesco Cerutti, Valeria Ghisetti, Stefano Bonora, Giovanni Di Perri, Andrea Calcagno

**Affiliations:** ^1^Unit of Infectious Diseases, Department of Medical Sciences, University of Torino at the “Amedeo di Savoia” Hospital, Turin, Italy; ^2^Microbiology and Molecular Biology Laboratory, “Amedeo di Savoia” Hospital, Turin, Italy

**Keywords:** SARS-CoV-2, COVID-19, viral inoculum, viral amount, cycle threshold, disease severity, secondary infections, outcomes

## Abstract

**Background:** Emerging evidence supports the “variolation hypothesis” in severe acute respiratory syndrome coronavirus-2 (SARS-CoV-2), but the derivative idea that the viral load of index cases may predict disease severity in secondary cases could be unsubstantiated. We assessed whether the prevalence of symptomatic infections, hospitalization, and deaths in household contacts of 2019 novel coronavirus disease (COVID-19) cases differed according to the SARS-CoV-2 PCR cycle threshold (Ct) from nasal-pharyngeal swab at diagnosis of linked index cases.

**Methods:** Cross-sectional study on household contacts of COVID-19 cases randomly sampled from all the infections diagnosed in March at our Microbiology Laboratory (Amedeo di Savoia, Turin). Data were retrospectively collected by phone interviews and from the Piedmont regional platform for COVID-19 emergency. Index cases were classified as high (HVl) and low viral load (LVl) according to two exploratory cut-offs of RdRp gene Ct value. Secondary cases were defined as swab confirmed or symptom based likely when not tested but presenting compatible clinical picture.

**Results:** One hundred thirty-two index cases of whom 87.9% symptomatic and 289 household contacts were included. The latter were male and Caucasian in 44.3 and 95.8% of cases, with a median age of 34 years (19–57). Seventy-four were swab confirmed and other 28 were symptom based likely secondary cases. Considering both, the contacts of HVl and LVl did not differ in the prevalence of symptomatic infections nor COVID-19-related hospitalization and death. No difference in median Ct of index cases between symptomatic and asymptomatic, hospitalized and not hospitalized, or deceased and survived secondary cases was found. Negative findings were confirmed after adjusting for differences in time between COVID-19 onset and swab collection of index cases (median 5 days) and after removing pediatric secondary cases.

**Conclusions:** The amount of SARS-CoV-2 of the source at diagnosis does not predict clinical outcomes of linked secondary cases. Considering the impelling release of assays for SARS-CoV-2 RNA exact quantification, these negative findings should inform clinical and public health strategies on how to interpret and use the data.

## Introduction

Since the beginning of severe acute respiratory syndrome coronavirus-2 (SARS-CoV-2) pandemic, a dose-response/effect relationship has been hypothesized between viral inoculum and 2019 novel coronavirus disease (COVID-19) severity, the “variolation” hypothesis (Van Damme et al., 2020). As for the principle of inoculation of small amount of smallpox in Variola immunization, people infected with a small amount of SARS-CoV-2 virions will on average develop asymptomatic or milder infections than people coming into contact with high viral loads, independently of other acknowledged risk factors of disease severity (Van Damme et al., 2020). According to this hypothesis, the contacts of cases that shed low viral loads will more likely develop a milder COVID-19 compared with secondary cases infected by index cases who spread higher doses (Van Damme et al., 2020). Indulging on this model, we could expect that secondary COVID-19 cases cluster in chains differentiated by disease severity with potential implications for public health interventions (Van Damme et al., 2020).

Increasing evidence supports a potential positive correlation between nasal-pharyngeal SARS-CoV-2 viral load at the time of care-seeking and both clinical manifestations and outcomes within an individual (Magleby et al., 2020; Rao et al., 2020). Applying these data to the previous framework could also lead to suggest considering severely affected people as a source of more cases and more severe infections, potentially requiring differential isolation strategies and management of infected contacts. However, to date, only one group described a differential severity of COVID-19 among three clusters likely acquiring the infection in three significantly different environments in terms of potential viral amount at the exposure (Guallar et al., 2020). The higher was the supposed initial viral exposure, the worse was COVID-19 severity (Guallar et al., 2020). In line with this, a review of the available evidence also supports the idea that the environmental exposure to SARS-CoV-2, meant as the product of the intensity and the duration of such exposure, has a positive correlation with the viral load detected within exposed and subsequently infected individuals as well as with the severity of the resulting COVID-19 (Calisti, 2020). Nevertheless, this conclusion cannot immediately translate the plausible association between inoculum and disease severity into an association between viral load of the index case and disease severity. In this regard, we were not able to find any study addressing the issue, despite hypotheses and media slogans.

Therefore, while the foundations of the “variolation” hypothesis are validly supported by comparisons with previous viral infections (Chu et al., 2004; Han et al., 2019; Little et al., 2020; Van Damme et al., 2020) and emerging evidence in COVID-19 (Faíco-Filho et al., 2020; Magleby et al., 2020; Pujadas et al., 2020; Rao et al., 2020; Shlomai et al., 2020; Van Damme et al., 2020; Westblade et al., 2020), several directly derivative ideas including that the viral load of index cases predicts disease severity in secondary cases could be inaccurate extrapolations.

Hence, to test the hypothesis that the higher the viral load is in index cases the more serious are the infections among linked secondary cases, we compared the prevalence of symptomatic infections, hospital admissions, and deaths due to COVID-19 between household cases secondary to index cases with known high and low diagnostic SARS-CoV-2 PCR cycle thresholds (Ct), as proxy of viral load.

## Materials And Methods

We performed a nested cross-sectional analysis on data retrospectively collected for an ongoing study on the relationship between diagnostic nasal-pharyngeal SARS-CoV-2 PCR Ct and risk of transmission among households.

Patients with a positive diagnostic nasal-pharyngeal SARS-CoV-2 swab performed at our Laboratory (Amedeo di Savoia Hospital, Turin, Italy) in March 2020 were randomly sampled (random lottery extraction) and reached in August–September for a phone interview addressing COVID-19-related clinical and demographic characteristics of both the interviewed and their household contacts, if any. The surveyed data were crosschecked and completed by data extrapolated by the Piedmont platform (RUPCOVID), an on-line regional database built up for SARS-CoV-2 contact tracing, notification (swab results and dates of index and contact cases), and clinical data collection (demographics, signs and symptoms at onset and at diagnostic swab, date of symptoms onset, comorbidities). In cases of data discrepancy between the phone interview and the RUPCOVID, the record reported in the database was considered and used. Patients not consenting to the phone survey were discarded and their data not collected from the RUPCOVID database. Anonymized data were used for survivor and deceased subjects. Patients that contracted the infection while already hospitalized for other reasons were excluded for the current report analysis. The study was approved by the Inter-company Department for Infectious Diseases and Emergency (DIRMEI, Torino, Italy).

Swab samples were processed by RT-PCR with the Novel Coronavirus (2019-nCoV) Real Time Multiplex RT-PCR kit (Liferiver Bio-Tech, San Diego, CA, United States; detection limit 1 × 10^3^ copies/mL), targeting three SARS-CoV-2 specific genes: RNA-dependent RNA Polymerase gene (RdRP), Nucleocapsid gene, and Envelope. For the purpose of the study, only RdRp gene Ct values were considered to have one uniform proxy of viral load, being RdRp the most specific gene among the three. The ABI Prism 7500 thermal cycler was used for PCR amplification (Thermo Fisher Scientific, Waltham, MA, United States). Ct is defined as the number of cycles of amplification required for the fluorescence of SARS-CoV-2 PCR to be detected above the background signal and can be used as a relative inverse proportional measure of viral amount in the specimen. The index cases were classified according to their diagnostic Ct using two different cut-offs:

A. A viral replication potential-based cut-off: ≤24.0 as high viral load (HVl), >24.0 as low viral load (LVl); 24 was chosen due to preliminary observations that the recovery of SARS-CoV-2 in Vero cell cultures inoculated by samples with PCR Ct >24 is significantly reduced if null (Corman et al., 2020; Jefferson et al., 2020).
B. A rapid antigen-based cut-off: ≤28.0 as HVl, >28.0 as LVl; 28 was chosen because of the 100% detection rate of SARS-CoV-2 rapid antigen in samples with a Ct <28, previously described (Cerutti et al., 2020).

Linked household COVID-19 cases were defined as secondary cases when the disease onsets at least 5 days after the manifestations of the index case, considering the lowest value of a recent pooled average incubation period estimate (Wassie et al., 2020); linked household asymptomatic cases were defined as secondary cases when the diagnostic nasal-pharyngeal swab was performed after the diagnosis of the index case only if a likely alternate source of infection for the index case was identified (as, for example, index cases acquiring the infection at work).

To define asymptomatic COVID-19 cases, at least one of the following signs and symptoms had to be reported: fever, asthenia, malaise, arthromyalgia, headache, olfactory and gustatory dysfunction, nausea, vomiting, diarrhea, dyspnea, runny nose, cough, and/or pharyngitis.

Hospitalization was considered COVID-19 related when due to the signs or symptoms described above or when no other reasons for hospital admission were reported (as, for example, occurrence of hyponatremia, atrial fibrillation, syncope, chest X-ray lesions in SARS-CoV-2-positive subjects).

Data were analyzed through nonparametric tests (Mann-Whitney *U* test, Chi-square for trend, Fisher’s exact test). Categorical variables are presented as absolute number (proportion), while continuous variables as median (interquartile range). Variables with relevant biological significance or showing univariate *p* ≤ 0.10 were included in the multiple ordinal logistic regressions (entry method). Data analysis was performed through SPSS 25.0 (IBM stat.).

## Results

### Index Cases

From the entire sample of 200 individuals included in the major study, 132 COVID-19 cases were alive at the survey (follow-up time of 6 months [6–7]) and lived with at least another person at the time of SARS-CoV-2 infection, so that data on household transmission was available and used for the present report.

In the index group, 79 subjects (59.8%) were male, 124 (93.9%) Caucasian, with a median age, Ct, and time from disease onset to diagnostic swab of 53 years (41–62), 30.54 (22.01–34.65; minimum 15.53 and maximum 39.18), and 5 days (3–10), respectively. One hundred and sixteen (87.9%) were symptomatic and 74 (56.0%) required hospital admission. According to 24 and 28 Ct cut-offs, 49 (37.1%) and 63 (47.7%) individuals were classified as HVl, respectively. The Ct groups did not differ for sex [Ct24: HVl 52 males (46.8%) vs. LVl 76 (42.7%), *p* = 0.49; Ct 28: HVl 55 males (41.0%) vs. LVl 73 (47.1%), *p* = 0.30] nor for age of household contacts [Ct 24: HVl 32 years (16–56) vs. LVl 35 (20–58), *p* = 0.80; Ct 28: HVl 36 years (18–58) vs. LVl 32 (20–56), *p* = 0.69]. The time from disease onset to diagnostic swab differed between the groups only when using 24 as cut-off [Ct 24: HVl 3 days (2–7) vs. LVl 7 (3–10), *p* = 0.030; Ct 28: HVl 4 days (2–8) vs. LVl 5 (3–10), *p* = 0.37].

### Secondary Cases

Overall, 289 were household contacts. Among them, 128 (44.3%) were male, 277 (95.8%) Caucasian, with a median age of 34 years (19–57). One hundred and sixty-seven (57.8%) underwent SARS-CoV-2 nasal-pharyngeal swab and were classified as certainly positive or negative accordingly; 103 (61.7%) were positive, of which 74 (44.3%) were linked secondary cases (median difference in time of COVID-19 onset between secondary and index cases of 6.5 days [5.5–8]). Of them, 67 (90.5%), 16 (21.6%), and 5 (6.8%) developed symptoms, required hospital admission, and died, respectively.

As for potential confounding, the age of household contacts stratified by whether the linked index case had high or low viral load did not differ when applying both the cut-offs: 53 years (32–70) vs. 52 years (30–64) and 59 years (40–69) vs. 47 years (29–63) in LVl vs. HVl for Ct 28 and 24, respectively (*p* = 0.953 and 0.355). Similarly, the sex of household contacts stratified by whether the linked index case had high or low viral load did not differ when applying both the cut-offs: 41.0 vs. 47.1% and 46.8 vs. 42.7% of male subjects in LVl vs. HVl for Ct 28 and 24, respectively (*p* = 0.301 and 0.489). On the contrary, the proportion of tested households differed between contacts of high and low viral load: 50.3 vs. 63.4% and 52.8 vs. 62.2% of subjects that underwent testing among contacts of LVl vs. HVl according to Ct 28 and 24, respectively (*p* = 0.025 and 0.119).

Among those not tested (122), 40 (13.8% of the overall contacts) had signs and symptoms suggestive of COVID-19 and were defined as likely positive. Of them, 28 (70.0%) developed the clinical picture after the onset of the linked index case (median time of 6 days [6–8]) and none of them required hospital admission nor died.

### Clinical Outcomes in Secondary Cases by Diagnostic Ct Value of Index Cases

**Figure 1:**
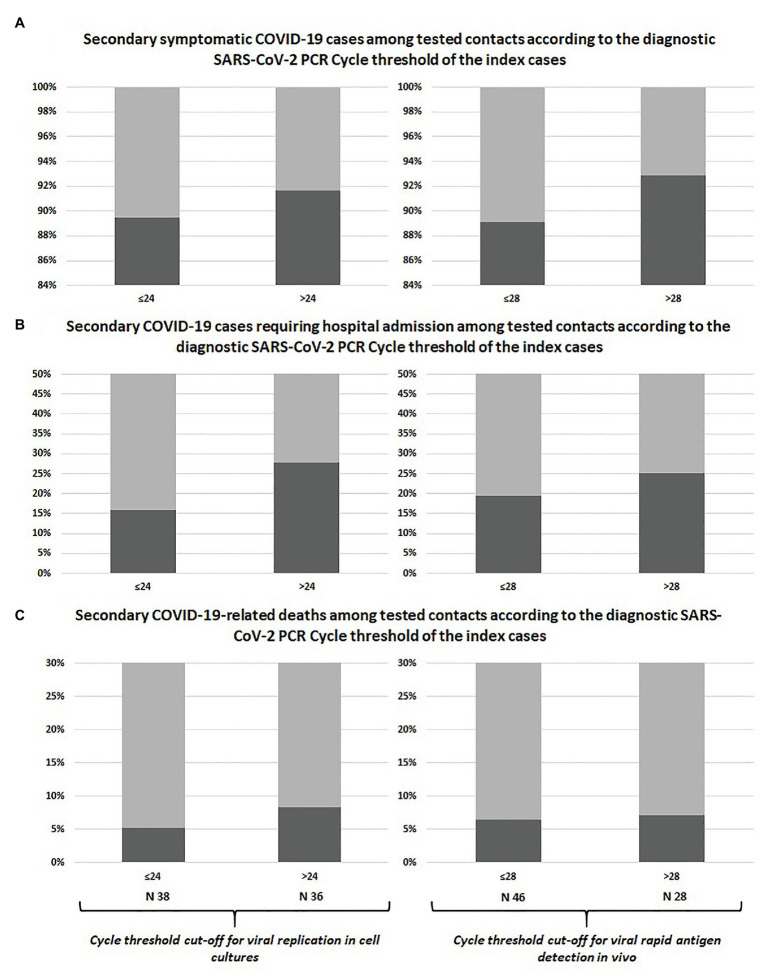
Comparison between swab-confirmed COVID-19 secondary cases of index cases with high vs. low viral load at diagnosis: symptomatic infections **(A)**, hospital admissions **(B)**, and deaths **(C)**.

**Figure 2:**
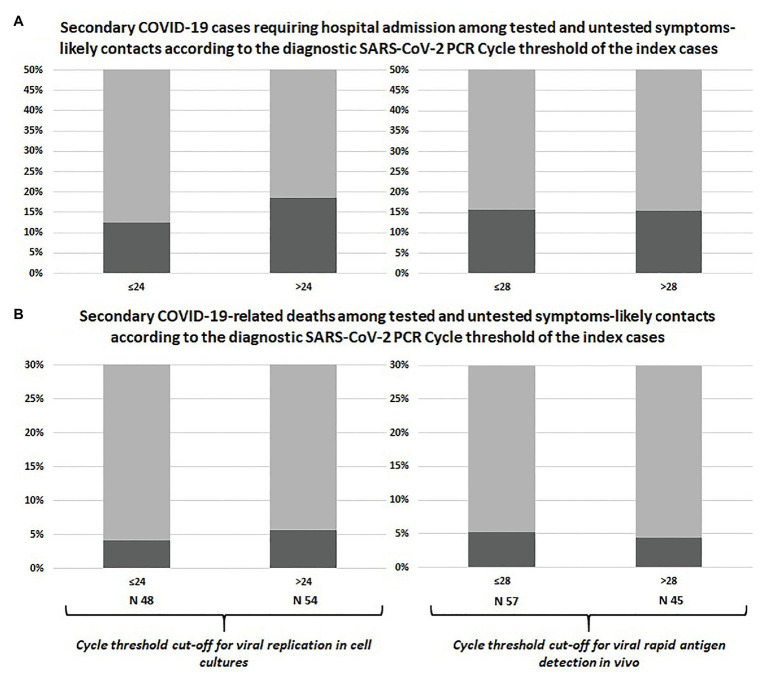
Comparison between swab-confirmed plus symptom-based likely COVID-19 secondary cases of index cases with high vs. low viral load: hospital admissions **(A)** and deaths **(B)**.

The prevalence of symptomatic infections, hospital admissions, and deaths among swab-positive secondary cases did not differ according to Ct of the index case classified with neither of the adopted cut-offs, as shown in Table 1 and Figure 1. Similar results were observed after including untested but likely positive individuals in the 2 × 2 comparisons (Table 1; Figure 2).

**Table 1:** Cross-tabulation for the differences in disease severity (symptoms, hospitalization and survival) according to SARS-CoV-2 PCR cycle threshold of the index case among swab-positive and swab-positive plus symptoms-based likely secondary cases.

	Viral replication cut-off			Rapid antigen detection cut-off		
**Swab-positive secondary cases (*n* = 74)**	**≤24 (*n*** = **38)**	**>24 (*n*** = **36)**	***p***	**≤28 (*n*** = **46)**	**>28 (*n*** = **28)**	***p***
Symptomatic	34 (89.5%)	33 (91.7%)	0.99	41 (89.1%)	26 (92.8%)	0.70
Asymptomatic	4 (10.5%)	3 (8.3%)		5 (10.9%)	2 (7.1%)	
Hospital admission	6 (15.8%)	10 (27.8%)	0.21	9 (19.6%)	7 (25.0%)	0.58
Home recovery	32 (84.2%)	26 (72.2%)		37 (80.4%)	21 (75.0%)	
Death	2 (5.3%)	3 (8.3%)	0.67	3 (6.5%)	2 (7.1%)	0.92
Survivor	36 (94.7%)	33 (91.7%)		43 (93.5%)	26 (92.9%)	
**Swab-positive plus likely positive secondary cases (*n*** = **102)**	**≤24 (*n*** = **48)**	**>24 (*n*** = **54)**	***p***	**≤28 (*n*** = **57)**	**>28 (*n*** = **45)**	***p***
Hospital admission	6 (12.5%)	10 (18.5%)	0.40	9 (15.8%)	7 (15.6%)	0.97
Home recovery	42 (87.5%)	44 (81.5%)		48 (84.2%)	38 (84.4%)	
Death	2 (4.2%)	3 (5.6%)	0.99	3 (5.3%)	2 (4.4%)	0.99
Survivor	46 (95.8%)	51 (94.4%)		54 (94.7%)	43 (95.6%)	
**Adult household contacts only**						
**Swab-positive secondary cases (*n*** = **65)**	**≤24 (*n*** = **33)**	**>24 (*n*** = **32)**	***p***	**≤28 (*n*** = **40)**	**>28 (*n*** = **25)**	***p***
Symptomatic	30 (%)	30 (%)	0.67	36 (%)	24 (%)	0.38
Asymptomatic	3 (%)	2 (%)		4 (%)	1 (%)	
Hospital admission	5 (%)	10 (%)	0.12	8 (%)	7 (%)	0.46
Home recovery	28 (%)	22 (%)		32 (%)	18 (%)	
Death	2 (%)	3 (%)	0.62	3 (%)	2 (%)	0.94
Survivor	31 (%)	29 (%)		37 (%)	23 (%)	
**Swab-positive plus likely positive secondary cases (*n*** = **91)**	**≤24 (*n*** = **43)**	**>24 (*n*** = **48)**	***p***	**≤28 (*n*** = **52)**	**>28 (*n*** = **39)**	***p***
Hospital admission	6 (%)	10 (%)	0.39	9 (%)	7 (%)	0.94
Home recovery	37 (%)	38 (%)		43 (%)	32 (%)	
Death	2 (%)	3 (%)	0.74	3 (%)	2 (%)	0.89
Survivor	41 (%)	45 (%)		49 (%)	37 (%)	

As a confirmation and to rule out potential arbitrariness of our Ct cut-offs, we evaluated the difference in median Ct values of the linked index cases between swab-positive only and swab-positive plus likely positive secondary cases grouped by the three outcomes (symptoms, hospitalization, and death): as reported in Table 2, once again there were no difference in the median Ct value of the linked index cases between symptomatic and asymptomatic, hospitalized and not hospitalized, as well as deceased and survived secondary cases.

**Table 2:** Comparison of median cycle threshold of the linked index cases between swab-positive and swab-positive plus likely positive household secondary cases grouped by clinical outcomes.

Swab-positive secondary household cases (*n* = 74)			
	Symptomatic (*n* = 67)	Asymptomatic (*n* = 7)	***p***
Index Ct value	24.68 (19.52–34.21)	22.02 (20.49–29.71)	*0.989*
	**Hospitalized (*n* = 16)**	**Not hospitalized (**n** = 58)**	***p***
Index Ct value	28.27 (17.93–35.63)	23.17 (19.56–31.39)	*0.397*
	**Dead (*n* = 5)**	**Survivor (*n* = 69)**	***p***
Index Ct value	28.66 (17.93–35.63)	24.63 (19.52–33.02)	*0.999*
**Swab-positive plus likely positive secondary household cases (*n* = 102)**			
	**Hospitalized (*n* = 16)**	**Not hospitalized (*n* = 86)**	***p***
Index Ct value	28.27 (17.93–35.63)	25.02 (19.97–34.05)	*0.721*
	**Dead (*n* = 5)**	**Survivor (*n* = 97)**	***p***
Index Ct value	28.66 (17.93–35.63)	26.14 (19.97–34.05)	*0.964*

Despite negative univariate findings, multivariate binary logistic regressions were run having the covariate Ct value of the index cases adjusted for the time gap between COVID-19 onset and the diagnostic swab collection to take into account potential changes in the nasal-pharyngeal viral load amount according to its dynamics. Even after correcting for this factor, no association was observed between the Ct value of the index case and the clinical outcomes of secondary cases, as shown in Table 3.

**Table 3:** Multivariate analyses: association between COVID-19-related symptomaticity, hospitalization and death in secondary cases and diagnostic Ct value of the index cases adjusted for relevant variables.

Variable	aOR	*p*
**Symptomatic COVID-19 in swab-positive secondary cases (R-squared 19.8%)**		
Ct value of the index case adjusted for the time from COVID-19 onset to the diagnostic swab	1.00 (0.99–1.01)	0.950
Age of secondary cases	1.07 (0.99–1.14)	0.055
Sex of secondary cases	3.60 (0.62–6.01)	0.126
**COVID-19-related hospitalization in swab-positive secondary cases (R-squared 26.3%)**		
Ct value of the index case adjusted for the time from COVID-19 onset to the diagnostic swab	1.00 (0.99–1.01)	0.625
Age of secondary cases	1.06 (1.02–1.10)	0.004
Sex of secondary cases	1.95 (0.57–6.62)	0.285
**COVID-19-related death in swab-positive secondary cases (R-squared 43.1%)**		
Ct value of the index case adjusted for the time from COVID-19 onset to diagnostic swab	0.99 (0.98–1.01)	0.306
Age of secondary cases	1.13 (1.01–1.26)	0.030
Sex of secondary cases	1.34 (0.15–11.74)	0.792
**COVID-19-related hospitalization in swab-positive plus likely positive secondary cases (R-squared 32.1%)**		
Ct value of the index case adjusted for the time from COVID-19 onset to the diagnostic swab	1.00 (0.99–1.01)	0.605
Age of secondary cases	1.07 (1.03–1.11)	<0.001
Sex of secondary cases	1.78 (0.53–5.97)	0.349
**COVID-19-related death in swab-positive plus likely positive secondary cases (R-squared 46.3%)**		
Ct value of the index case adjusted for the time from COVID-19 onset to the diagnostic swab	0.99 (0.98–1.01)	0.308
Age of secondary cases	1.14 (1.02–1.27)	0.021
Sex of secondary cases	1.23 (0.14–10.75)	0.851

### Sensitivity Analysis by Age

Sixty-seven household contacts (23.1%) were below 18 years of age; as this part of the population seems to be at lower risk of symptomatic and/or severe infection, we performed a sensitivity analysis by repeating the previous comparison after removing this group of individuals. As shown in the lower sections of Table 1, the prevalence of symptomatic infections, hospital admissions, and deaths in swab-positive only and swab-positive plus likely positive secondary cases did not differ according to Ct of the index case even when the analysis was restricted to adult subjects only.

## Discussion

We did not observe any difference in disease severity in terms of symptoms, hospitalization requirement, and survival among secondary cases according to the nasal-pharyngeal viral load of the index case in a setting representative of household transmission dynamics at the beginning of the COVID-19 epidemic in Piedmont, one of the most heavily afflicted regions of Italy. To the best of our knowledge, this is the first report assessing potential associations between a proxy of the nasal-pharyngeal SARS-CoV-2 viral load of index cases at diagnosis and COVID-19 severity in secondary cases.

Among the limitations of our study, indeed the sample size is one. Nevertheless, the higher prevalence of the worst outcomes among secondary cases exposed to LVl compared with those exposed to HVl leave us more confident in not rejecting our null hypotheses despite the lower powerfulness of nonparametric tests to reach significance. Furthermore, collecting and analyzing real-life data to test our hypothesis may be challenging as it requires prominent resources and efforts to control for such a large amount of variables, that heavily limits possibilities as well as available sample size.

The considerably long follow-up time (median 6 months) limited the amount of collected data and the accuracy of the information about symptoms. Nevertheless, the aim of our study was the preliminary assessment of a rough association between viral load of index cases and relevant overall clinical outcomes of their linked secondary cases. Furthermore, such a long follow-up assures us about the reliability of survival and hospitalization, which could have been otherwise biased by observational periods concluding at hospital or emergency department discharge and missing potential rehospitalization or subsequent deaths. Lastly, while recall bias may have affected symptoms reporting, it is highly unlikely that they had any effect upon the other clinical outcomes considered (survival, hospitalization) and data that may be roughly affected by accurate memory were all double checked and corrected based on the official records reported by the RUPCOVID platform.

Diagnostic Ct value may not always be representative of the highest viral load experienced by index cases during the period of potential transmission, but the relatively short time from disease onset to the first swab of our sample (median 5 days) reassures us about the timeliness and representativeness of the Ct snapshot and classification of the index cases. Indeed, the viral peak in the upper respiratory airways has been described as occurring from few hours before or on symptoms onset to the first week of overt disease with subsequent decay that differs in its rapidity according to several factors (He et al., 2020; Singanayagam et al., 2020; Zheng et al., 2020). We did not observe any association between the Ct of index cases and the analyzed clinical outcomes at multivariate analysis, even after accounting for potential differences in the delay of swab collections from the onset of COVID-19, which should have mitigate the potential effect of viral dynamics upon the reliability of the Ct as an early proxy of the highest amount of virus in the index case.

We have no available data on comorbidities of households, but the median age of the household contacts was young (34 years) and the eventual inclusion of comorbidity in the model would have plausibly increased the goodness of fit of the regressions, without altering the significance. Besides, the number of comorbidities per patient significantly correlated with age (rho 0.48, *p* < 0.001). Despite we could have not specifically adjusted our analyses for the comorbidities of secondary cases, the inclusion of contacts’ age may have still partially take into account this factor.

We have also no data regarding protective measures adopted within the households nor the characteristics of the home environment; however, as for the former, the sample belongs to the first initial period of the pandemic in our country, so that it should represents a naïve population with scarce adoption of protective measures and immunity. The high prevalence of symptomatic infections among contacts that underwent testing could be explained by the tendency at the beginning of the pandemic to test more rapidly and commonly only subjects reporting symptoms due to the scarcity of resources during the emergency. We attempted to correct this bias including in the second part of the analyses also untested households reporting suggestive signs or symptoms, observing similar results. Lastly, we could not rule out that some of the secondary cases contracted the infection outside the household, especially for the positives within the first weeks, having the lockdown started in the 7th of March.

While aleatory uncertainty is inevitable and constant, epistemic uncertainty is still at its peak as for the emerging SARS-CoV-2 infection. Hypotheses and models are formulated at the same pace as the speed of newly notified COVID-19 cases, and the lack of data or the discrepancy between the available ones do not set limits to theories. Epistemology could also be misleading: infective dose, viral inoculum, and viral load of the source are sometimes used interchangeably in the current debate, although they may not indicate the same thing even in experimental models.

Quantitative assays for the exact determination of SARS-CoV-2 are under development and will be soon available for routine practice, aiming at improving both the clinical management of patients and containment strategies. Facing new diseases requires an effort to clarify gap of knowledge that may be dangerously given for grant not to misuse tools and resources. The affirmation that contacts of COVID-19 cases spreading higher amount of virus are at higher risk of more severe infections is intriguing but not so plausible and could lead to unnecessary anxiety among the general population as well as to differential clinical strategies that may prove ineffective.

**Figure 3:**
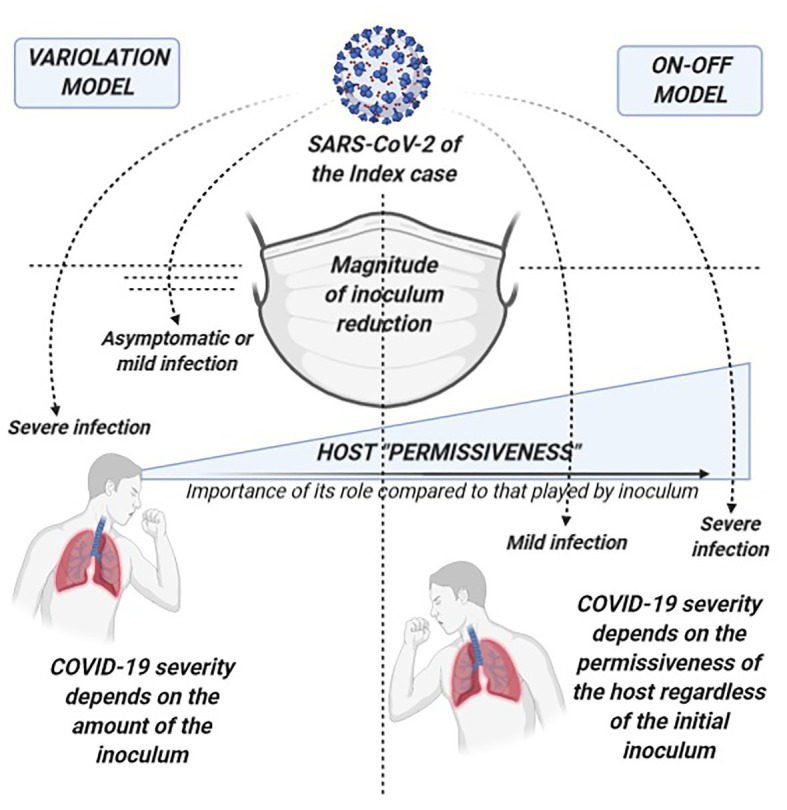
Comparison of the potential of self-protective measures in the “variolation” vs. the “on-off” model for the relationship between COVID-19 severity and viral inoculum. On the left, the variolation model applied to the potential role of self-protective measures (such as facial masks) in COVID-19 severity of secondary cases: this model presupposes that the viral inoculum plays a significant role in the subsequent immune pathology of the infection and in the final clinical outcomes. Therefore, COVID-19 severity will significantly depend also on the amount of virus that can break through protective measures such as masks, delineating a clinical scenario where the more efficient is the virion filtration made by face masking the milder will be the disease, other known severity determinants being equal. On the right, the on-off model where our data better fit. On-off refers to the fact that the trigger to the immune-pathogenesis and subsequent clinical outcomes rely on the presence or absence of the virus rather than on a graded scale of its amount. Regardless of the entity of the viral exposure, it is the host “permissiveness” to viral replication and pathogenicity (represented by age, sex, receptor density, genetic and epigenetic factors, host immunological features, comorbidities, comedications, etc.) that leads the clinical evolution of SARS-CoV-2 infection. While host permissiveness has the same weight as viral inoculum in determining disease severity in the variolation model, in the on-off model, it is the major driver and determinant of disease severity, overwhelming what could be the contribution of viral inoculum. In this scenario, the role of protective measures is also on-off as it relies on the complete abrogation in acquiring the infection with little or no impact on COVID-19 severity through the modulation of the amount of the virus.

An example of this is represented by the current debate on whether self-protective measures such as facial masks, that are undoubtedly able to reduce SARS-CoV-2 transmission and new infections, might also reduce COVID-19 severity among people who get infected by decreasing the amount of viral inoculum (Brosseau et al., 2020; Gandhi and Rutherford, 2020a,b; Rasmussen et al., 2020). Animal models report conflicting evidence on the supposed dose-response relationship in SARS-CoV-2 infection (Munster et al., 2020; Ryan et al., 2021). Furthermore, emerging mutant variants affecting replication and infectivity potential have not yet been proved to cause more severe diseases (Korber et al., 2020). A comparison of the impact on COVID-19 severity from the proper application of self-protective measures within the context of the variolation model and of an alternative on-off model that tries to explain our negative findings is depicted in Figure 3. Our results would be better explained by a pathogenic model where the presence of SARS-CoV-2 and subsequently the effect of self-protective measures act both according to an on-off mechanism. Rather than the amount of virus which a subject is exposed to, it seems to be the host permissiveness to the subsequent local and systemic viral replication as well as the permissiveness to trigger inflammatory and immunologic processes that significantly drive the clinical evolution of the infection. As a consequence, it seems to be the presence or absence (on-off) of SARS-CoV-2 per se, regardless of the initial viral inoculum, combined with the host permissiveness that determines COVID-19 severity. Accordingly, the usefulness of self-protective measures depends on completely abrogating the viral transmission or not (on-off), rather than modulating the amount of viral inoculum.

**Figure 4:**
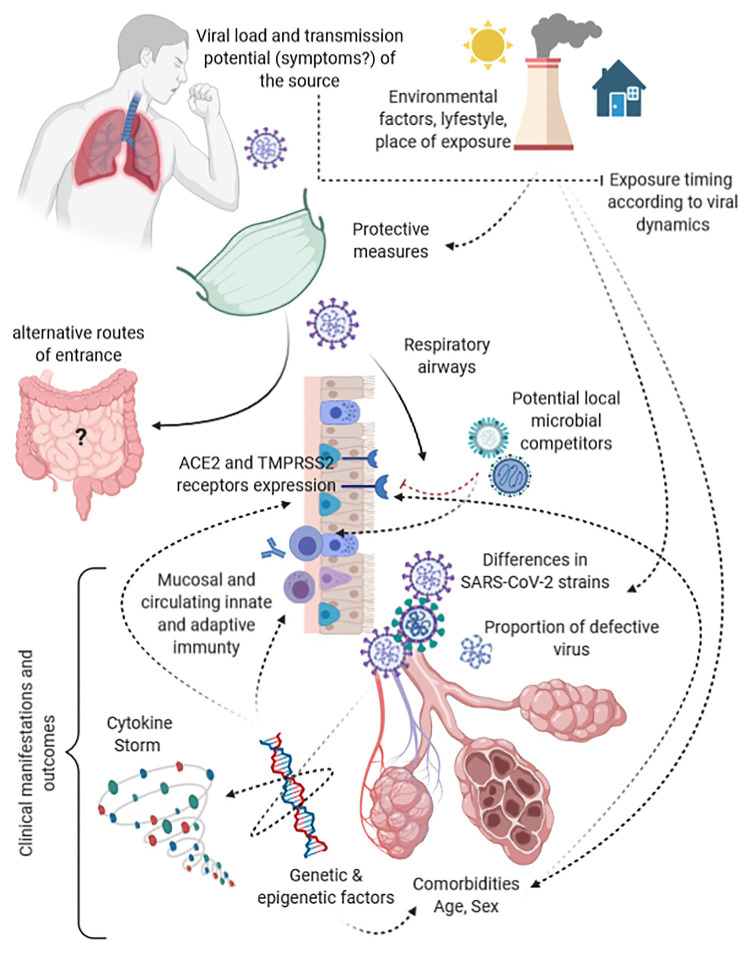
Funnels and modulators along the path from the viral load of the index case to the target receptors and clinical outcomes in secondary cases.

This hypothesis is not supported by our preliminary data only but is in line with two other emerging observations. First, symptomatic and asymptomatic patients have shown to be frequently characterized by similar amount of virus at the beginning of the infection (Long et al., 2020; Louie et al., 2020). Therefore, underlying determinants other than the amount of SARS-CoV-2 should explain this divergent evolution in spite of a similar amount of virus. These determinants constitute what we have called permissiveness, both to viral replication and to ignition of pathogenic mechanisms, and are represented by several variables such as age, sex, comorbidities, comedications, and immunological, genetic, and epigenetic factors (as shown in Figure 4). Secondly, confusion surrounds the terminology used by studies assessing COVID-19 severity and the amount of virus at diagnosis. Once again, what has been found to predict COVID-19 severity is not the viral inoculum but the amount of virus that is locally replicating since at least few days before detection, as the shorter median period from disease onset to swab collection among the studies is 5 days (de la Calle et al., 2021; Trunfio et al., 2021). No evidence is yet available on the quantitative change from the amount of the viral inoculum to the amount of detected virus at symptom onset nor at diagnosis to support or reject this hypothesis, but the epistemological difference is substantial.

As supported by our negative observation, despite the mounting evidence in favor of a predictive and prognostic value of the early amount of SARS-CoV-2 viral load carried by an infected person in terms of disease severity and transmission potential of the same person (Basile et al., 2020; Binnicker, 2020; Faíco-Filho et al., 2020; Magleby et al., 2020; Pujadas et al., 2020; Rao et al., 2020; Shlomai et al., 2020; Singanayagam et al., 2020; Van Damme et al., 2020; Westblade et al., 2020), the same variable may not be a useful factor to be taken into account in predicting potential clinical outcomes of linked secondary COVID-19 cases. Indeed, this hypothesis does not take into account several factors acting as a funnel between the viral load of sources and the virions reaching the mucosal receptors and eventually causing clinical manifestations in secondary cases (Figure 4). Moreover, it does not consider pathogenic mechanisms of SARS-CoV-2, the likelihood that this infection acts both locally and at distance from the entry site, host, and viral genetic and epigenetic factors intertwined with immune modulators underling the cytokine storm, viral adaptation strategies, and intra- and interviral species competition (Domingo et al., 2020; Jacob, 2020; Kuri-Cervantes et al., 2020). All of these are potentially able to impact upon the covariance between viral load of the source, the eventual inoculum, and the resulting disease severity. As example, ACE2 and TMPRSS2 receptor density is significantly affected by genetics, comorbidities, drugs, age, and type of tissue, probably representing one of the most impactful bottleneck and permissiveness determinant in the SARS-CoV-2 path from the virus of the source to the infective virus involved in the eventual dose-response/effect (Bao et al., 2020; Gheblawi et al., 2020; Jacob, 2020). This long cascade of determinants acting as funnels and filters make extremely different the virus spread by the source, the virus reaching a receptive subject, and the virus eventually causing the disease, highlighting a likely uselessness of the viral load of human sources among the determinants that have to be taken into account in predicting and modeling clusters of symptomatic or severe disease.

In conclusion, the contacts of a carrier with high viral load could be at higher risk of acquiring the infection, but to date, there is no evidence that the acquired infection will be more likely to be more symptomatic or severe. Our pilot study, taking into account the several acknowledged limitations, does not support similar theories that would require further data before being embraced to permeate public health strategies and public opinion.

## Data Availability Statement

Raw and derived data supporting the findings of this study are available from the corresponding author (MT) on request.

## Ethics Statement

The studies involving human participants were reviewed and approved by The Regional Inter-company Department for Infectious Diseases and Emergency (DIRMEI, Torino, Italy). Written informed consent to participate in this study was provided by the participants’ legal guardian/next of kin in the event of impossibility to give consent by participants themselves.

## Author Contributions

MT, SB, GP, and AC: study design and ideation. MT, BL, FA, FV, FC, and VG: data collection. MT, FC, VG, and AC: data analysis. MT, FC, VG, SB, GP, and AC: interpretation of results and critical appraisal. MT, GP, and AC: draft writing. MT, BL, FA, FV, FC, VG, SB, GP, and AC: manuscript revision. All authors contributed to the article and approved the submitted version.

### Conflict of Interest

The authors declare that the research was conducted in the absence of any commercial or financial relationships that could be construed as a potential conflict of interest.
